# Novel variant of *FBN2* in a patient with congenital contractual arachnodactyly

**DOI:** 10.1038/s41439-024-00264-1

**Published:** 2024-02-08

**Authors:** Mina Nakama, Yuki Miwa, Sayaka Manabe, Shigeru Shimamoto, Hidenori Ohnishi

**Affiliations:** 1 Department of Life Science, Faculty of Science and Engineering, Kindai University, Osaka, Japan; 2 Department of Pediatrics, Graduate School of Medicine, Gifu University, Gifu, Japan; 3 Clinical Genetics Center, Gifu University Hospital, Gifu, Japan

**Keywords:** Genetic testing, Genetic testing

## Abstract

Congenital contractual arachnodactyly (CCA) is a genetic connective tissue disorder that is characterized by arachnodactyly, kyphoscoliosis, marfanoid habitus, and crumpled ears. We report a case of a boy with suspected Marfan syndrome. Genetic analysis revealed c.3207_3217+9del in a heterozygote form of the fibrillin-2 (*FBN2)* gene. This patient was diagnosed with CCA based on his phenotype, and the pathogenicity of this variant was classified according to cDNA analysis and protein modeling.

Congenital contractual arachnodactyly (CCA) is a genetic connective tissue disorder. Patients with CCA tend to have a distinctive Marfan-like appearance and present with joint contractures, arachnodactyly, crumpled ears, and kyphoscoliosis^1^. Early diagnosis and follow-up of this disorder ensure that patients can refrain from physical contact sports or activities and LASIK eye surgery^2^. In particular, it is important to differentiate CCA from Marfan syndrome (MFS), which has cardiovascular involvement^1^. CCA is caused by pathogenic variants in the *FBN2* (OMIM#121050) gene, which is located on chromosome 5q23.3. It encodes fibrillin-2, which is a large extracellular matrix protein that multimerizes into linear structures called microfibrils. These calcium-binding structures have intrinsic elasticity and can associate with elastin to form elastic fibers. Microfibrils provide support for both elastic and nonelastic connective tissues, are involved in cell-matrix communication, and contribute to extracellular growth factor sequestration and regulation. Fibrillin-2 is homologous to fibrillin-1, which is encoded by *FBN1* (of which pathogenic variants cause MFS), but it is mainly expressed during early embryonic development. Notably, the disease-associated variants in *FBN2* are located in the central region of the gene (exons 24–35), which encode for a central stretch of calcium-binding epidermal growth factor-like (cbEGF-like) domains^3^.

We examined a case of a 14-year-old boy with a height of 177.4 cm, an arm span of 179.5 cm, and a weight of 51.9 kg, with long slim limbs, long spider-like fingers, and scoliosis. He had a positive wrist sign and a positive thumb sign on his left hand. No ectopia lentis or myopia had been observed thus far, although the patient was suspected to have MFS. His heartbeat was normal, and he did not have the typical abnormally crumpled ears. Informed consent for molecular analysis was obtained from the patient, and this study was approved by the Ethics Committee of Gifu University Hospital (approval reference number 2018-181). A gene panel analysis that targeted connective tissue diseases using next-generation sequencing was performed at the Kazusa DNA Research Institute. The target genes were *FBN1*, *FBN2*, *TGFBR1*, *TGFBR2*, *SMAD3*, *TGFB2*, *TGFB3*, *ACTA2*, *MYH11*, *MYLK*, *COL3A1*, *EFEMP2*, *FLNA*, and *SLC2A10*. Genomic DNA was extracted from the leukocytes of the patient’s peripheral blood. Total RNA was isolated from the patient’s fresh blood sample and a fibroblast sample (Kurabo, Osaka, Japan) as a healthy control using an Isogen kit (Nippon Gene, Tokyo, Japan). Sanger sequencing was performed using the 5′-GCATCTTGCCCACCAGTACA-3′ (forward) and 5′-CATCTCCGGTTGCAGAGGAA-3′ (reverse) primers. Reverse transcription was performed using the *FBN2*-specific antisense primers Ex24RTr (5′-AAGAACATCCCCTCGGTTAGC-3′), Ex26RTr (5′-GAGGTCAGGAGAAATCCTGC-3′), and an oligo (dT) primer (Thermo Fisher Scientific, Waltham, MA, USA). For the amplification of cDNA, a forward primer for exon 21 (5′-CTGAAATCTGAATGCTGTGCC-3′) and a reverse primer for exon 26 (5′-GAGGTCAGGAGAAATCCTGC-3′) were used. Conservation analysis of the variant was performed in *Mus musculus* (house mouse) NM_010181.2, *Rattus norvegicus* (Norway rat) NM_031826.2, *Bos taurus* (cattle) NM_001278588.1, *Canis lupus familiaris* (dog) XM_038681416.1, and *Pan troglodytes* (chimpanzee) XM_016953727.2. SpliceAI was used for the mutation prediction. For the fibrillin-2 protein, the structures of the wild-type and variant were predicted using the SWISS-MODEL automated protein homology modeling server (http://swissmodel.expasy.org).

Genetic analysis of the patient’s sample revealed a novel heterozygous deletion of *FBN2* genomic description NC_000005.10:g.128345351_128345370del. This variant is not present in population databases (gnomAD and jMorp). We referred to ClinVar_20230820 to determine whether the variant identified in the patient was novel. To the best of our knowledge, this variant has not yet been reported as a responsible mutation for CCA in the Japanese population or elsewhere. In addition, this variant was confirmed by Sanger sequencing (data not shown). We performed cDNA analysis because the patient’s genomic variant included a splicing site and in silico data indicated the predominant splice donor loss within his transcripts in SpliceAI. The patient’s RNA revealed two bands: the wild-type and a fragment that was shorter than the control (Fig. 1a). Although we did not check the relative expression levels of these two PCR products, the shorter band appears to be more abundant. Direct sequencing of the shorter PCR fragment from the patient showed a deletion (108 bp) at the end of exon 24, which resulted in an r.3110_3217 deletion (Fig. 1b). This is predicted to cause the deletion of 36 amino acids and substitutions in the FBN2 protein (p.(Cys1037_Lys1073delinsTyr)) but preserves the integrity of the reading frame (Supplementary Fig. 1). This large deletion of amino acids in fibrillin-2, including an antiparallel β-sheet, may cause structural protein folding instability in the model (Fig. 2) and the loss of the RGD (arginine-glycine-aspartic acid) sequence. Because RGD is a common sequence for integrin binding, which is important for cell adhesion^4^, the variant is predicted to decrease cell adhesion ability. Moreover, this variant is present in the TB3-cbEGF11 domain, where another pathogenic missense mutation, p.Gly1057Asp, was previously reported^5^. This variant is also located in a hotspot for most CCA-causing mutations^3^. The amino acid sequence of this variant position is highly conserved among species, which indicates that this region may play an important role in fibrillin-2 (Supplementary Fig. 2). There is possible for other forms of *FBN2* transcripts in the patient because we performed RT-PCR in only exons 21–26, which can be considered as a limitation of our analysis. His unaffected parents did not approve genetic testing for themselves. Therefore, this variant was not confirmed to be de novo. The variant was classified as likely pathogenic according to the American College of Medical Genetics and Genomics guidelines^6^. The pathogenicity criteria fulfilled the PM1 (pathogenic, moderate), PM2 (pathogenic, moderate), and PM4 (pathogenic, moderate) categories.

**Fig. 1:**
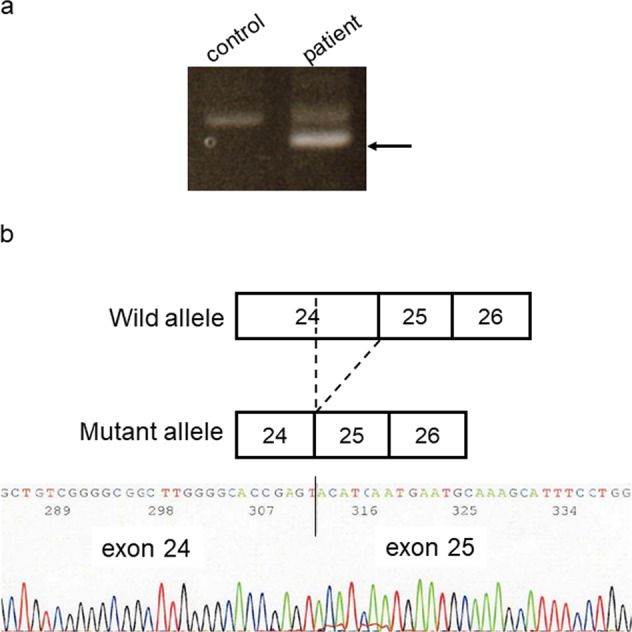
Representation of RT-PCR results in the mis-splicing of exon 24. **a** Agarose gel electrophoresis of the RT-PCR product, the arrow indicates the shorter band of the patient sample. Transcripts from the control fibroblasts showed only the predicted wild-type amplicon size of 630 bp. **b** Schematic representation of the abnormal *FBN2* transcripts. The line indicates the partial sequencing of the exon 24 exon 25 boundary of the mutant allele cDNA. The cDNA sequencing of the aberrant amplification band revealed a truncated exon 24.

**Fig. 2:**
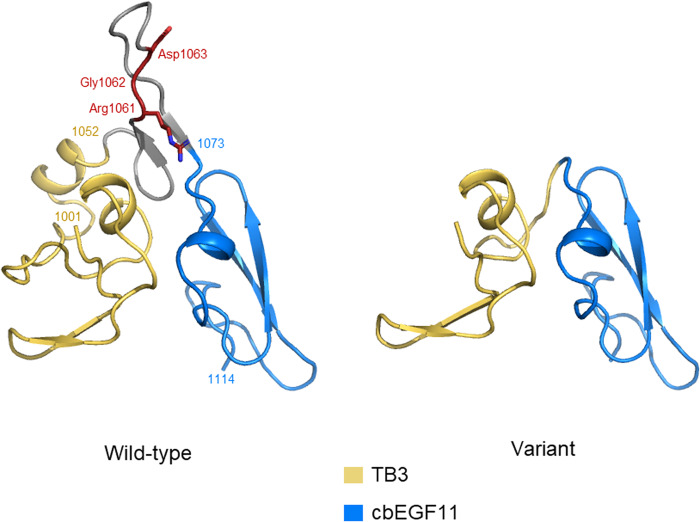
Modeled structure of the TB3 and cbEGF11 domains of fibrillin-2. Modeled structures of the TB3-cbRGF11 domains of the FBN2 protein are illustrated in the wild-type and variant forms. TB3 is colored yellow, cbEGF11 is colored blue, and the RGD sequence is colored red.

To conclude, the present study reports a novel heterozygous non-canonical *FBN2* variant, c.3207_3217+9del, which was identified in a Japanese patient with CCA. Considering all the factors together, including the patient phenotype, transcript alterations, and protein modeling, the variant was suggested as a pathogenic variant that resulted in CCA.

## HGV Database

The relevant data from this Data Report are hosted at the Human Genome Variation Database at 10.6084/m9.figshare.hgv.3362.

## Supplementary information

Supplementary Figure 1

Supplementary Figure 2

## Data Availability

The data described in this report are available upon request. Supplementary Information is available online through the Human Genome Variation.

## Materials availability

The materials described in this report are available upon request. Supplementary Information is available online through the Human Genome Variation.
