# Supplementary figures and images for: Novel variant of FBN2 in a patient with congenital contractual arachnodactyly
Source: Hum Genome Var. 2024 Feb 8;11:7. doi: 10.1038/s41439-024-00264-1 (PMC10850470; doi:10.1038/s41439-024-00264-1)

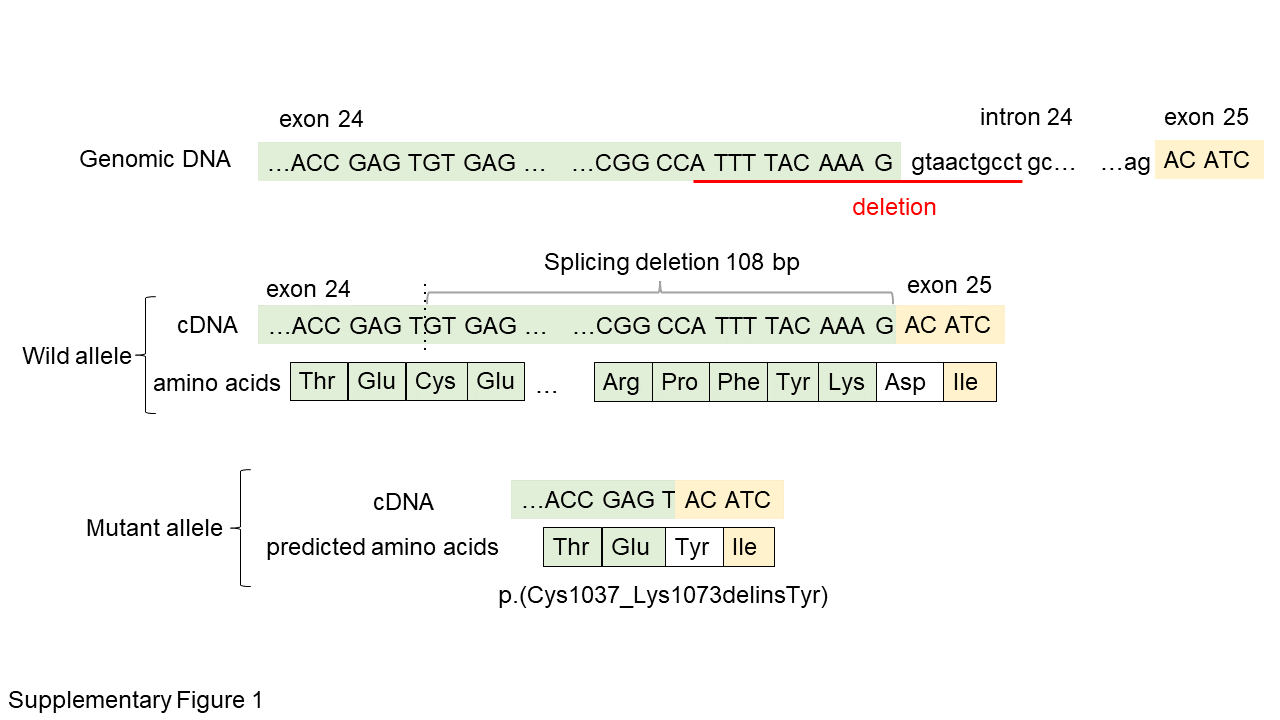

Supplement: Supplementary file 1 — Supplementary Figure 1 [file 41439_2024_264_MOESM1_ESM.png]

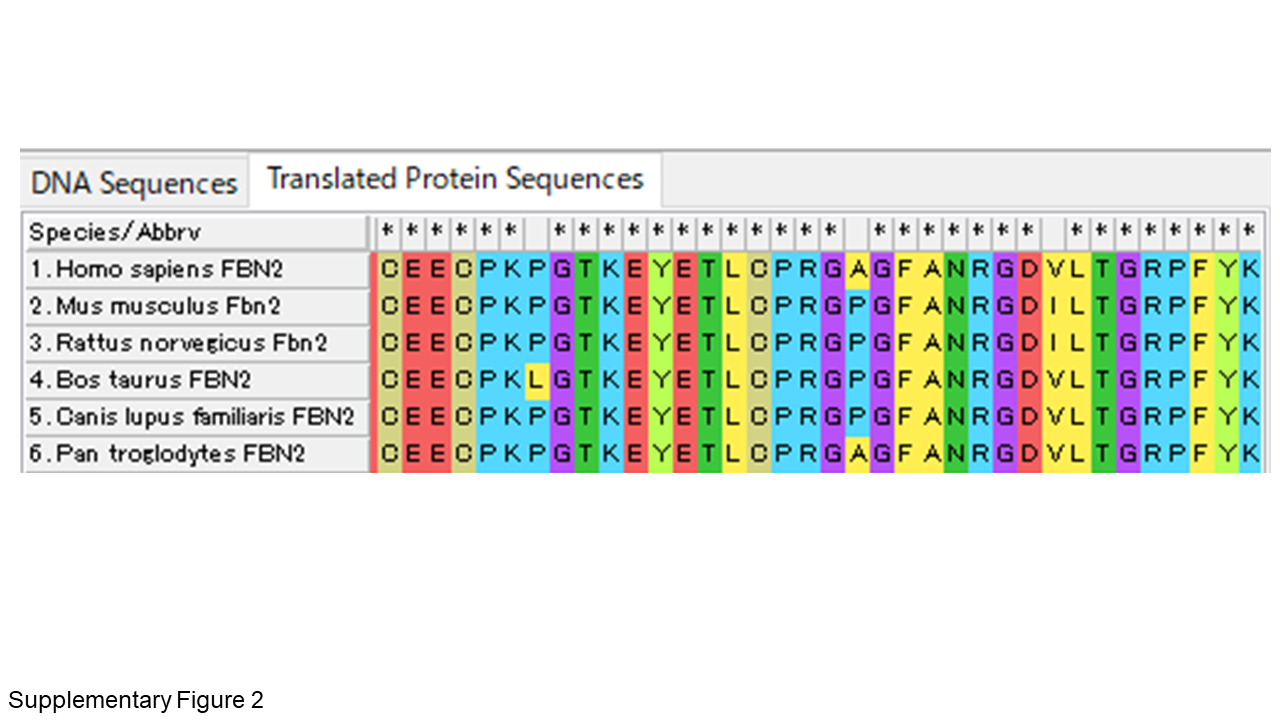

Supplement: Supplementary file 2 — Supplementary Figure 2 [file 41439_2024_264_MOESM2_ESM.png]
